# Discovery of Crystallizable Organic Semiconductors with Machine Learning

**DOI:** 10.1021/jacs.4c05245

**Published:** 2024-07-25

**Authors:** Holly M. Johnson, Filipp Gusev, Jordan T. Dull, Yejoon Seo, Rodney D. Priestley, Olexandr Isayev, Barry P. Rand

**Affiliations:** † Department of Electrical and Computer Engineering, Princeton University, Princeton, New Jersey 08544, United States; ‡ Computational Biology Department, School of Computer Science, Carnegie Mellon University, Pittsburgh, Pennsylvania 15213, United States; ¶ Department of Chemistry, Mellon College of Science, Carnegie Mellon University, Pittsburgh, Pennsylvania 15213, United States; § Department of Chemical and Biological Engineering, Princeton University, Princeton, New Jersey 08544, United States; ∥ Andlinger Center for Energy and the Environment, Princeton University, Princeton, New Jersey 08544, United States

## Abstract

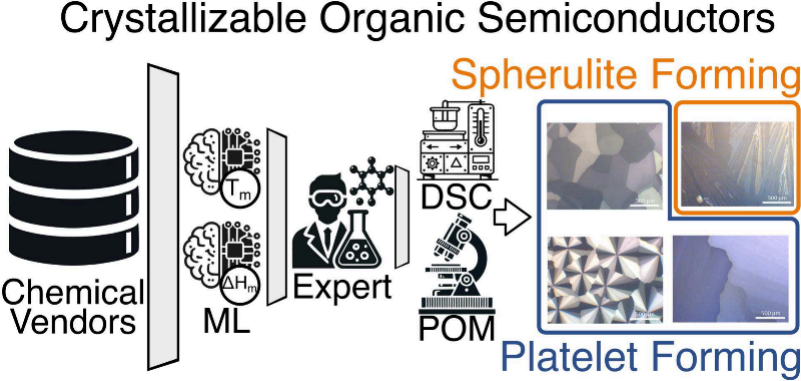

Crystalline organic semiconductors are known to have improved charge carrier mobility and exciton diffusion length in comparison to their amorphous counterparts. Certain organic molecular thin films can be transitioned from initially prepared amorphous layers to large-scale crystalline films via abrupt thermal annealing. Ideally, these films crystallize as platelets with long-range-ordered domains on the scale of tens to hundreds of microns. However, other organic molecular thin films may instead crystallize as spherulites or resist crystallization entirely. Organic molecules that have the capability of transforming into a platelet morphology feature both high melting point (*T*_*m*_) and crystallization driving force (*ΔG*_*c*_). In this work, we employed machine learning (ML) to identify candidate organic materials with the potential to crystallize into platelets by estimating the aforementioned thermal properties. Six organic molecules identified by the ML algorithm were experimentally evaluated; three crystallized as platelets, one crystallized as a spherulite, and two resisted thin film crystallization. These results demonstrate a successful application of ML in the scope of predicting thermal properties of organic molecules and reinforce the principles of *T*_*m*_ and *ΔG*_*c*_ as metrics that aid in predicting the crystallization behavior of organic thin films.

## Introduction

Thin film devices composed of organic semiconductors (OSCs) have gained significant attention due to their compatibility with large area deposition, optoelectronic tunability, and mechanical flexibility.^1^ Organic photovoltaic cells (OPVs) and organic light-emitting diodes (OLEDs) have been the most prominent; OPVs have recently reached efficiencies as high as 19.2%,^2^ while OLEDs have gained public acceptance in the display sector.^3^ Typically, organic thin films are amorphous when incorporated into a device, despite crystalline organic films featuring improved exciton diffusion length^4,5^ and charge carrier mobility,^6^ often by several orders of magnitude. There are several methods for crystallizing organic thin films, including adding polymer or small-molecule additives to OSC solutions,^7^ solvent vapor annealing,^8^ organic epitaxial growth,^9^ and abrupt thermal annealing.^10−15^ In this work, we employed an abrupt thermal annealing technique to achieve organic thin-film crystallization.

Amorphous organic thin films fabricated via vacuum thermal deposition can transition into crystalline films upon annealing. The morphology of the crystals that are grown with this technique depend on the molecule itself and the experimental conditions used during fabrication. These conditions are found through experimental optimization and focus on factors such as the thickness of the organic layer, the presence of an organic underlayer, and annealing conditions, among other considerations.^10,11^ There are different crystalline morphologies that can result from this optimization, such as platelets or spherulites. From the point of view of electronic devices, it is more favorable to have a thin film crystallize as a platelet as spherulites have worse charge carrier mobility compared to platelet crystals^11^ and contain many voids and pinholes that can lead to shunting and compromise device yield. Spherulite crystals can exhibit multiple morphologies, such as smooth gradients stemming from a single nucleation point or as sharper, needle-like crystals. Platelet crystals are large-area single crystals on the scale of tens to hundreds of microns across. The long-range order and few grain boundaries minimize deleterious effects such as exciton recombination^16^ or carrier scattering.^12,13,17^

However, identifying OSCs that crystallize in this manner is not straightforward, making the pursuit of crystalline organic electronics challenging. Recently, we reported that the thermal properties of OSCs correlate with crystallization in either a platelet or spherulite morphology; the thermal properties guiding these trends are the material’s crystallization driving force (*ΔG*_*c*_) and melting point (*T*_*m*_).^10^ Materials with high *ΔG*_*c*_ and high *T*_*m*_ have a tendency to crystallize as platelets, and as such we can use these values as a guide for selecting organic molecules. Despite its usefulness as a predictive factor, *ΔG*_*c*_ is not a readily available value in databases containing information on OSC molecules and crystal structures, meaning that the determination of the value of *ΔG*_*c*_ is still necessary.

To overcome these limitations, we employed machine learning (ML) to predict the thermal properties of OSCs, specifically *T*_*m*_ and *ΔG*_*c*_. The application of ML to OSCs in previous literature has focused on analyzing and predicting traits such as charge carrier mobility,^18,19^ thermal conductivity,^20^ static and dynamic disorder as it applies to charge transport,^21,22^ and vibrational thermal characteristics such as entropy, specific heat, and dielectric function.^23^ In reference to devices, ML has been used to predict the power conversion efficiency (PCE) of OPVs^24^ and to aid in the design of more efficient OLEDs, such as by identifying thermally activated delayed fluorescent (TADF) emitters.^25^ The application of virtual screening, originally popularized in the drug discovery field,^26−28^ has found its use in other branches of chemical sciences.^29−31^ Here, we applied descriptor-based ML models to screen commercially available virtual libraries for putative platelet-forming OSCs suitable for experimental validation. We experimentally assessed six organic molecules identified by the virtual screening and were able to crystallize three of these into films with large-area platelets, validating the ML predictions and reinforcing that thermal properties correlate to crystallization behavior.

## Results and Discussion

**Figure 1:**
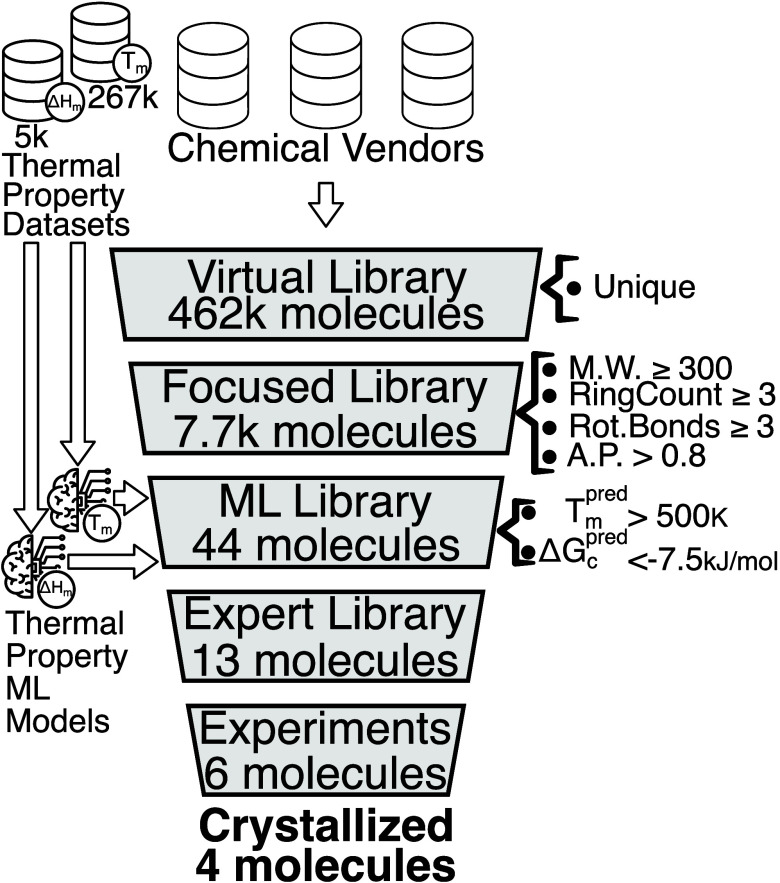
General scheme of the screening campaign: filters and ML-models applied on the corresponding stage of virtual screening shown are described in the text and SI.

A schematic of our virtual screening is shown in Figure 1, where several databases comprised of ≈462,000 commercially available organic molecules served as the starting point. In a number of subsequent steps, the database was screened for a combination of favorable chemical properties. Various factors were then taken into account to filter out materials incompatible with experimental constraints. Organic materials with a molecular weight (MW) of less than 300 g/mol were not considered, as materials with low MW have high vapor pressures that can linger in and contaminate vacuum chambers. Other experimental factors considered included the composition of the organic molecules, the number of rotatable bonds, the number of conjugated rings, and the aromatic proportions in each molecule that can give insight into its behavior as a potential crystallizing material and semiconductor. Having too many rotatable bonds inhibits crystallization, while having too few rotatable bonds is also not advantageous, as such molecules may crystallize upon deposition rather than in a post-deposition annealing step. Hence, we looked at materials that had at least three rotatable bonds (Figure 1), which has been shown previously to be the start of the platelet-forming region for organic molecules,^10^ and limited the number of aromatic cycles by excluding fullerene-like molecules. We filtered the pool further by selecting molecules with three or more conjugated rings, which, due to the π-electron delocalization from p-orbital overlap caused by alternating single and double bonds, lends to semiconducting behavior.^32,33^

Application of these constraints reduced the number of candidate organic molecules to 7,742, forming a focused library. From here, ML modeling was employed to predict the *T*_*m*_ and *ΔG*_*c*_ of these molecules. We define *ΔG*_*c*_ as
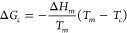
1

where *T*_*c*_ is the material’s cold crystallization temperature and *ΔH*_*m*_ is the enthalpy of melting.

The focused library (Figure 1) was further screened by ML models trained on thermal properties (See SI-1.2 Model development for details and https://github.com/isayevlab/Discovery_COS_wML for trained models, training data and descriptor lists) for molecules with predicted *T*_*m*_ > 500 K and *ΔG*_*c*_ < −7.5 kJ/mol. This resulted in 44 candidates (see Table S3) belonging to the platelet-forming region. From these 44, the pool was narrowed down to 13 through consideration of commercial availability and price. Retrospectively, we also evaluated the pipeline’s ability to recover those molecules that were previously shown to crystallize as platelets. Out of 6 reported platelet materials,^10^ 4 were present in the virtual library. All 4 passed to the focused library stage.

**Figure 2:**
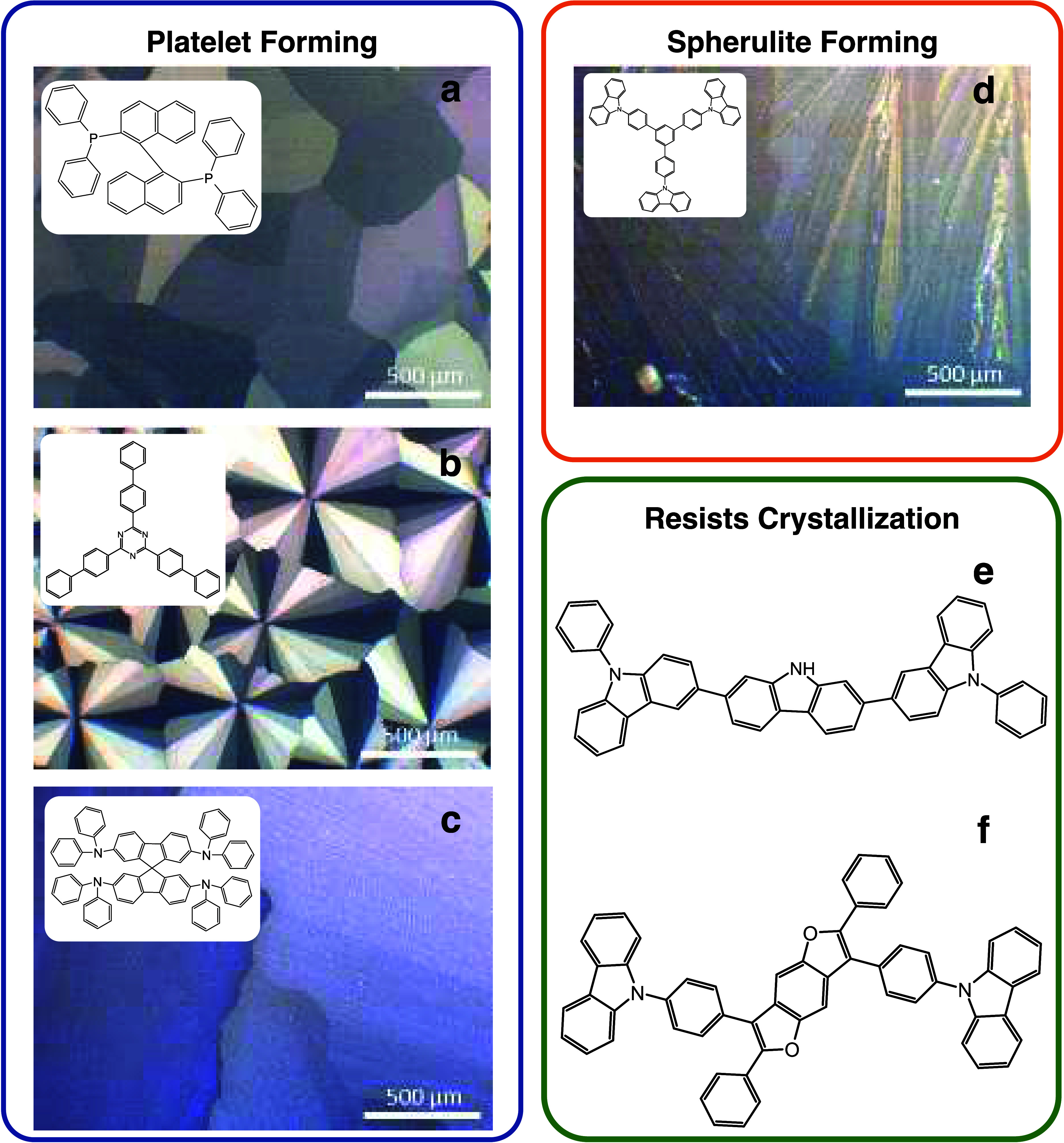
Polarized optical microscopy (POM) images inset with molecular structures of the materials used in this work, grouped by morphology. Here, (a) rac-BINAP, (b) TBT, and (c) spiro-TAD crystallized as platelets; (d) TPB-Cz crystallized as a spherulite; and (e) 9DT and (f) CZBDF resisted crystallization. The letters labeling each material correspond with the letters used in Table 1.

Of these 13 identified molecules, 6 were chosen that fit the experimental parameters, had a molecular structure that aligned with device-building and crystal-forming qualities, and had high predicted values of *T*_*m*_ and *ΔG*_*c*_. These six were rac-BINAP, TBT, spiro-TAD, TPB-Cz, 9DT, and CZBDF. Full chemical names are included in SI-1.4 Materials. See molecular structures in Figure 2.

Bulk differential scanning calorimetry (DSC) was performed on each material (Figure S2) to determine thermal properties for comparison to predicted values. The predicted values of *T*_*m*_ and *ΔG*_*c*_ and the experimentally derived thermal properties, *T*_*m*_, *T*_*c*_, and *ΔH*_*m*_, are reported in Table 1. Taking the onset values of these properties, we calculated an experimental value of *ΔG*_*c*_ as expressed by eq 1,^10^ also reported in Table 1.

**Table 1:**
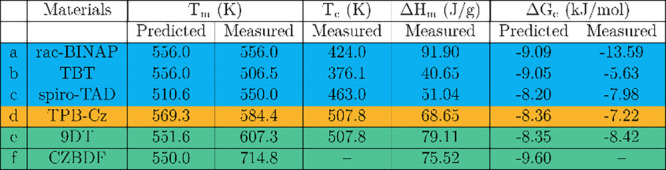
Predicted and Experimentally Derived Thermal Properties of Materials in This Work^a^

a The experimental values were determined via DSC and based on the onset values of each thermal event. The DSC scan of CZBDF had no crystallization peak upon heating, explaining the absence of an experimental *T*_*c*_ and calculation of Δ*G*_*c*_.

The DSC scan of TBT required further interpretation to calculate *ΔG*_*c*_. The initial DSC scans of the as-received TBT showed multiple endothermic events. To gain a better understanding of the thermal behavior of this material, TBT was deposited onto a glass substrate in an effort to get rid of potential impurities and form an amorphous thin film. This film was scraped off to form a powder that was then used for DSC. In iterative rounds of heating and cooling, we saw two melting peaks during the first heating (Figure S1b), an indication that the material was not fully amorphous upon deposition and a possible sign of polymorphism,^34^ and in following heating scans only the second peak appeared. As the first melting peak was only accessible from the as-deposited TBT, and that is the material used to fabricate and crystallize the thin film, we used the onset of the first melting peak to calculate the *ΔG*_*c*_ of TBT.

The initial DSC scans of CZBDF were featureless, with no thermal events up to 673.0 K (Figure S 2f). To further analyze this material, a separate DSC system was used to rerun CZBDF at a higher temperature, recorded in Figure S3. This scan showed an onset melting peak at 714.8 K, which was quite far from the ML predicted *T*_*m*_ of 550.0 K. Crystallization was observed upon cooling, but not upon heating, so *ΔG*_*c*_ could not be calculated.

The process to probe thin film crystal motifs involves annealing films of various thickness at temperatures at and above their glass transition temperature (*T*_g_) as determined through DSC.^10,11^ In the cases of TBT and spiro-TAD, 5 nm thick organic underlayers were added to aid crystallization.^11^ These conditions are reported in Table S4. Polarized optical microscopy (POM) was performed on these films after annealing to determine morphology and coverage. The POM images of the optimized thin-film crystal growth for these six molecules are shown in Figure 2, grouped together by the type of crystallinity exhibited after optimization. Three of the six molecules investigated crystallized as platelets: rac-BINAP (Figure 2a), TBT (Figure 2b), and spiro-TAD (Figure 2c). The optimized rac-BINAP film displayed full-coverage platelet domains. The next molecule, TBT, crystallized in a unique manner with multiple single crystal domains growing outward from a single nucleation point reminiscent of spherulitic crystal growth. However, due to the limited number of crystal domains and the clear distinction between each domain, this motif is still considered a platelet. The last platelet-forming material, spiro-TAD, had the largest platelet crystal domains studied here, on the scale of millimeters. Only TPB-Cz crystallized as a spherulite (Figure 2d), with visibly rough, sharp, and needle-like crystals with many domains growing from the same nucleation point. Two molecules resisted crystallization: 9DT (Figure 2e) and CZBDF (Figure 2f). The first, 9DT, showed no evidence of crystallization through numerous optimizations, despite its DSC scan showing a clear glass transition and crystallization peak upon heating (Figure S2e). This phenomenon has been observed in a molecule we have previously investigated, di-[4-(N,N-di-p-tolylamino)-phenyl]cyclohexane (TAPC), which also resisted crystallization despite the presence of a crystallization peak in DSC.^10^ The second material to resist crystallization, CZBDF, had an inconclusive POM (Figure S1a), so to confirm if the film was crystalline, X-ray diffraction (XRD) was performed on the annealed films. The XRD pattern showed one peak at 2θ = 30.2°, corresponding to the ITO substrate,^35^ and no other peaks to indicate crystal growth (Figure S1b). Due to this, CZBDF was also defined as a molecule that resists crystallization. It is notable that a melting peak of CZBDF could not be observed until 714.8 K. This can give us further insight into the limits of the range of platelet forming materials as the melting point of CZBDF is significantly higher than the platelet-forming materials we have studied previously. As the *T*_*m*_ of CZBDF fell significantly out of the range of the other platelet forming molecules and the material was difficult to characterize, it was considered an outlier and excluded from further analysis.

**Figure 3:**
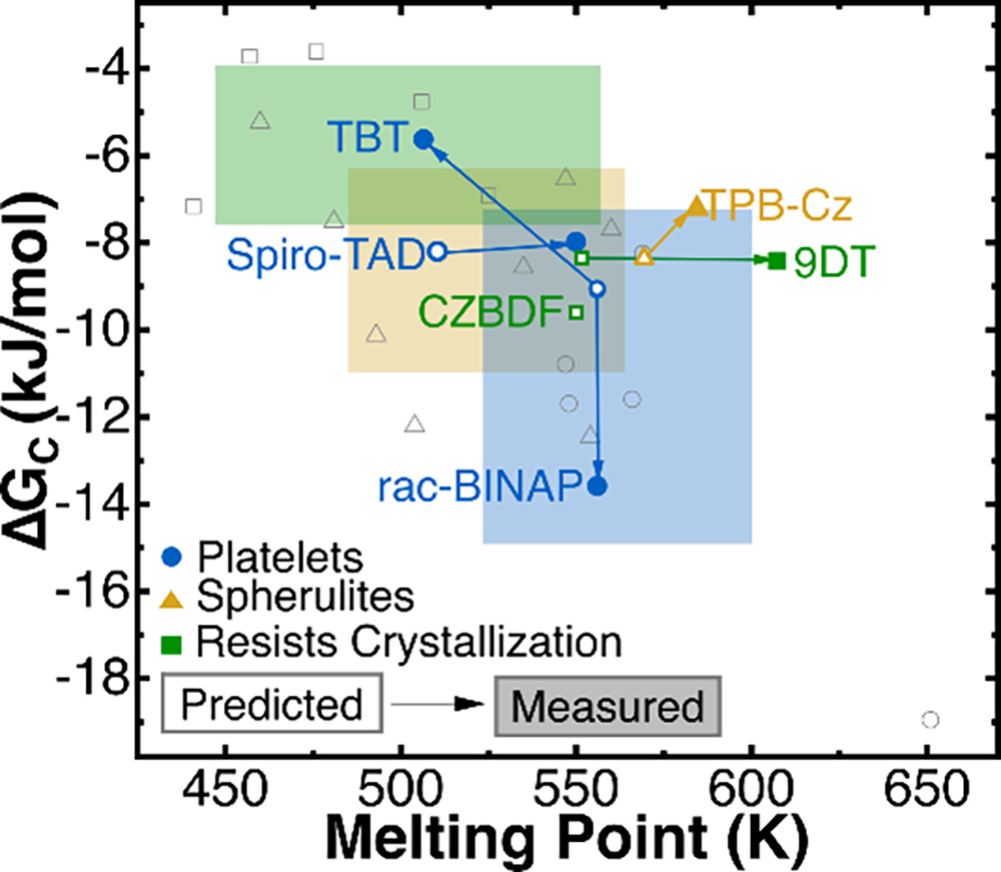
Predicted and measured crystallization driving force at *T*_*c*_ (*ΔG*_*c*_) as a function of *T*_*m*_. The arrows on each line point from the ML prediction (empty symbols) to the experimental findings (filled symbols). All other data on this graph come from previous experimental findings^10^ and are included in the calculations of the shaded regions, representing the experimental average and one standard deviation of *ΔG*_*c*_ and *T*_*m*_ associated with each morphological trend. Experimental data for CZBDF is not included as its *ΔG*_*c*_ could not be determined.

With these materials classified into their morphologies, we then combined these results with our previously reported thermal properties of 22 other organic molecules,^10^ as shown in Figure 3 and color coded according to whether they form platelets, spherulites, or resist crystallization. The shaded regions represent the average and one standard deviation of the cumulative *T*_*m*_ and *ΔG*_*c*_ for each respective crystallization category. Each prominent data point shows the predicted and experimental *T*_*m*_ and *ΔG*_*c*_ for the six materials studied in this work and are shaped and color-coded to indicate the morphology of that particular molecule. The empty symbols indicate the ML predicted values, while the filled symbols represent experimental values. Lines guide the eye from the predicted to experimental results, with the exception of CZBDF which only uses the predictions. The 22 previously reported molecules^10^ are included but greyed out, with the symbol corresponding to the crystal morphology.

The *T*_*m*_ and *ΔG*_*c*_ of platelet-forming organic molecules have been reported to be higher on average than the *T*_*m*_ and *ΔG*_*c*_ of spherulites, and markedly higher than organic molecules that resist crystallization,^10^ though these regions did overlap and contain outliers. For example, TBT was predicted to fall into the average range of a platelet crystal, yet its experimentally measured thermal properties sited it in a region where we would expect it to resist crystallization. Even so, TBT successfully crystallized as a platelet. While there is not a confirmed cause for this deviation, it is possible that there exists a physical factor not currently considered in this model that affects crystallization. In the case of TBT, this material showed signs of polymorphism in its DSC scan, and polymorphism is not a factor that is accounted for in this model. Investigation into the effects of these types of physical characteristics on crystallization will be the subject of future work. Similarly with 9DT, its predicted and experimentally derived *T*_*m*_ and *ΔG*_*c*_ fell within range of spherulitic or platelet growth, yet its film resisted crystallization. These regions, while valuable in producing trends of crystal morphology based on *T*_*m*_ and *ΔG*_*c*_, are not strict boundaries, and further work is needed to fully understand if there are other reasons for exceptions.

We note that the prediction of *T*_*m*_ for organic molecules is a substantial challenge, and that there is a range of accuracy in the predicted *T*_*m*_ and *ΔG*_*c*_ when compared to the measured thermal properties. The models created in this study for *T*_*m*_ and *ΔH*_*m*_ work under the assumption that all the entries in each dataset referenced are correct, without any means to accurately verify this assumption. The overall root mean square error (RMSE) in fully characterized materials is 38.3 K, which is in line with prior work, as the current state-of-the-art predictive models^36^ have RMSEs of 30-40 K. The model accuracy is limited by the extreme heterogeneity of the *T*_*m*_ data, decomposition upon melting, polymorphism in organic crystals, and amorphous forms. Such information is missing from current databases, and represent areas where improvements can be made.

**Figure 4:**
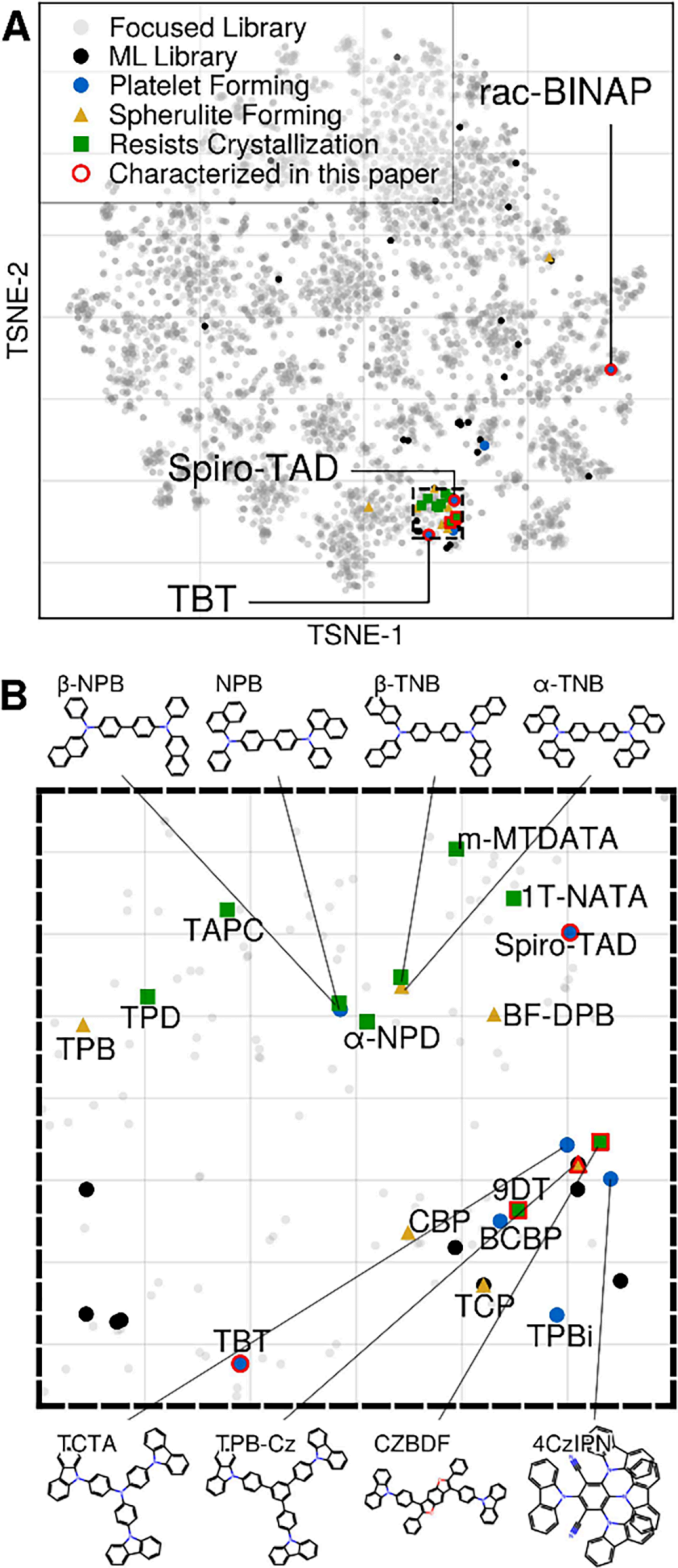
(A) The tSNE projection of the Focused Library and ML Library (cf. Figure 1). The crystal morphology is annotated for previously^10^ characterized materials as well as those investigated in this work. (B) Zoom-in subspace highlighted by the dashed frame in (A).

To highlight the diverse crystallization behavior observed, we performed a visualization of the relevant chemical space using the Focused library (cf. Figure 1) and all known crystallizable organic semiconductors. Figure 4a shows a 2D t-distributed stochastic neighbor embedding (tSNE) projection of 7,742 candidate molecules. Known materials are indicated with different colors depending on their crystallization behavior and morphology. Most of the known materials are localized in a tight cluster (Figure 4b). Rac-BINAP, discovered in the present work, expands this space to phosphorus-containing molecules. Even inside the known space (Figure 4b), two newly crystallized molecules, TAD and TBT, pushed the boundaries of known compounds.

Figure 4 presents an orthogonal projection of the data in Figure 3. However, it conveys a more complicated story of morphology classification. There is a very delicate balance between the molecular structure and crystal morphology. For example, between four closely related molecules (NPB, β-NPB, α-TNB, β-TNB, see Figure 4b), NPB forms a platelet, α-TNB is a spherulite-forming material, and the rest resist crystallization. Another interesting cluster is formed by carbazole derivatives (TCTA, TPB-Cz, CZBDF, 4CzIPN) where only two are platelet forming materials, highlighting the complexity of the structure-crystallization property landscape.

The use of ML predictions and expert knowledge work in tandem to identify platelet-forming candidate materials. The ML algorithm significantly narrows down the pool of molecules to consider to a size that makes it possible to assess all the remaining molecules individually, and expertise in crystallization allows us to further filter this pool by removing molecules we know intuitively will not result in desired outcomes. In the 44 molecules making up the ML library (Figure 1), there were 3 molecules we know from previous work to crystallize: rubrene and TCTA as platelets, and TCP as a spherulite,^10^ demonstrating successful hits of the ML algorithm (Table S3). There were also 2 molecules in this library that we know resist crystallization, α-sexithiophene and p-sexiphenyl (Table S3), owing to the fact that they will crystallize upon initial deposition. While the ML algorithm identified these 2 molecules as potential platelet-forming organic materials, utilizing experimental knowledge allowed us to identify them as misses. Combining the ML predictions with experimental expertise makes it possible to identify potential hits while avoiding misses, highlighting the necessity of both in identifying platelet-forming organics.

## Conclusion

We have demonstrated the successful application of ML in the scope of organic crystallization. Using ML, we screened nearly half a million commercially available organic molecules and selected several dozen that fit within the necessary property-composition space. Of these, six molecules were chosen based on qualities that aligned with what previous experimentation has shown to lead to organic crystalline thin films. Three of the six molecules crystallized as large-area platelets, one crystallized as a spherulite, and two resisted crystallization. This displayed a significant success rate of 50% in identifying organics that can crystallize as long-range-ordered platelet domains, the ideal form for future device applications, via the post-deposition annealing method. The work presented here enhances our understanding of the mechanisms and physical characteristics that affect thin film crystallization while underscoring challenges in predictions of thermal properties for organic crystals. Both *T*_*m*_ and *ΔH*_*m*_ are important not only for organic semiconductors but also have significant implications for pharmaceutical development and product formulations.

Machine learning is still a nascent field in its application to organic materials, hence, it is difficult to put current success in full context. However, the success rate substantially surpassed that of drug discovery virtual screening efforts, which rely on the guidance of experienced medicinal chemists for selection of molecules to bind a specific protein.^37,38^ In our study, we demonstrated that well-trained ML models are capable of predicting thermal properties and replicating the expertise of experimentalists in choosing molecules for validation. This represents an opportunity for a significant shift in decision-making authority from human experts to algorithms. These capabilities mark a crucial advancement towards self-driving laboratories, illustrating the collaborative potential of machine and human intelligence.

## Methods

### Computational

Datasets of *T*_*m*_ and *ΔH*_*m*_ were compiled from multiple sources and preprocessed independently according to a method published previously.^39^ The *T*_*m*_ dataset included in total ≈267,000 molecules, while the *ΔH*_*m*_ dataset contained ≈5,000 molecules (SI-1.1 Datasets preparation for virtual screening).

Two Gradient Boosting Decision Trees (XGBoost) based machine learning models were developed for *T*_*m*_ and *ΔH*_*m*_ using molecular descriptors for featurization. Trained models achieved mean absolute error (MAE) 32 ± 1 K and MAE 6.7 ± 0.2 kJ/mol for *T*_*m*_ and *ΔH*_*m*_ in 5-fold-cross-validation (SI-1.2 Model development).

A chemical library of 462,000 commercially available molecules was virtually screened for key chemical properties, reducing it to 7,742 molecules. This focused library was screened further with ML models, leading to the selection of 44 candidate molecules suitable for expert assessment (SI-1.3 Virtual screening). For t-SNE analysis, molecules were featurized as a superset of molecular descriptors used for *T*_*m*_ and *ΔH*_*m*_ ML-models (SI-1.4 t-SNE analysis).

Full details of the computational methods are included in the SI.

### Experimental

All materials studied in this work were purchased from commercial vendors and used as received (SI-1.5 Materials). Thin films were fabricated via vacuum thermal deposition and post-deposition annealing in an inert environment (SI-1.6 Fabrication).

Analysis of the materials’ thermal properties was done via DSC. Determination of the films’ crystalline quality was done via POM and, when necessary, XRD (SI-1.7 Equipment and characterization).

Full details of the experimental methods are included in the SI.

## Supporting Information Available

The supplemental information contains full descriptions of the computational and experimental methods, including dataset preparation for virtual screening, details of the model development and virtual screening, t-SNE analysis, a full list of materials and vendors, fabrication details, experimental equipment, and characterization techniques; supplemental data including DSC scans of the six materials studied here; POM, XRD, and secondary scans of CZBDF; *T*_*m*_ ML model performance; *ΔH*_*m*_ ML model performance; lists of the AlvaDesc’s molecular descriptors used for building both the *T*_*m*_ and *ΔH*_*m*_ ML-model; a complete summary of the focused ML library; and the fabrication conditions used to optimize crystal growth. Data, code, and ML models are available at https://github.com/isayevlab/Discovery_COS_wML (PDF)
