# Supplementary material for: Discovery of Crystallizable Organic Semiconductors with Machine Learning
Source: J Am Chem Soc. 2024 Jul 25;146(31):21583–90. doi: 10.1021/jacs.4c05245 (PMC11311223; doi:10.1021/jacs.4c05245)
Supplement: Supplementary file 1 — ja4c05245_si_001.pdf [file ja4c05245_si_001.pdf]

Supporting Information: Discovery of Crystallizable Organic Semiconductors with Machine Learning

Holly M. Johnson,[†,⊥] Filipp Gusev,[‡,¶,⊥] Jordan T. Dull,[†,⊥] Yejoon Seo,[§] Rodney D. Priestley,[§] Olexandr Isayev,[*,¶,‡] and Barry P. Rand[*,†,‖]

†*Department of Electrical and Computer Engineering, Princeton, New Jersey 08544, United States*

‡*Computational Biology Department, School of Computer Science, Carnegie Mellon University, Pittsburgh, Pennsylvania 15213, United States*

¶*Department of Chemistry, Mellon College of Science, Carnegie Mellon University, Pittsburgh, Pennsylvania 15213, United States*

§*Department of Chemical and Biological Engineering, Princeton, New Jersey 08544, United States*

‖*Andlinger Center for Energy and the Environment, Princeton, New Jersey 08544, United States*

⊥*These authors contributed equally to this work.*

E-mail: olexandr@olexandrisayev.com; brand@princeton.edu

Contents

SI-1 Methods **S-4**

SI-1.1 Datasets preparation for virtual screening S-4

SI-1.2 Model development S-4

SI-1.3 Virtual screening S-30

SI-1.4 t-SNE analysis S-30

SI-1.5 Materials S-32

SI-1.6 Fabrication S-32

SI-1.7 Equipment and characterization S-33

References **S-38**

List of Figures

S1 POM and XRD of CZBDF S-34

S2 DSC of all the materials studied in this work S-35

S3 Secondary DSC scan of CZBDF S-36

S4 T_m ML model performance S-36

S5 ΔH_m ML model performance S-37

List of Tables

S1 List of AlvaDesc's molecular descriptors used for building T_m ML-model . . . S-6

S2 List of AlvaDesc's molecular descriptors used for building ΔH_m ML-model . S-21

S3 Focused ML Library S-31

S4 Optimized fabrication conditions for the crystallized materials in this work . S-33

SI-1 Methods

SI-1.1 Datasets preparation for virtual screening

To enable the virtual screening for crystallizable OSCs, we performed independent modeling of two chemical properties, the melting temperature (T_m) and the enthalpy of melting (ΔH_m). To achieve this, we compiled datasets from diverse sources and preprocessed them independently. The melting temperature dataset was assembled from the United States Patent and Trademark Office (USPTO) patent records[S1] containing measured T_m for approximately 220 thousand molecules and T_m data from Tetko *et al.*[S2] of approximately 47 thousand molecules. Experimentally measured fusion (melting) enthalpy data were sourced from Acree *et al.*[S3,S4,S5] leading to a dataset of approximately 5 thousand molecules. Melting temperature (T_m) and the enthalpy of melting (ΔH_m) data were combined across both thermal properties independently, and duplicated records were treated according to Fourches *et al.*[S6]. Molecular structures, represented in SMILES format, were standardized using MolVS[S7].

SI-1.2 Model development

In this study, two machine learning models were developed based on the datasets for T_m and ΔH_m independently. Molecules were featurized by computing descriptors available through AlvaDesc 2.0[S8]. The preprocessing of molecular descriptors was conducted using the Scikit-learn library[S9] according to the following protocol independently for each dataset. First, each individual descriptor was scaled via MinMax scaler. Second, scaled descriptors with a variance of less than 0.01 were filtered out. Third, for the remaining subset of descriptors pairwise spearman correlation matrix was computed and only one descriptor from each pair of cross-correlated (descriptors with absolute spearman correlation of more than 0.9) was kept, leading to creation of preprocessed dataset suitable for modelling. Preprocessed dataset consisted of 476 descriptors (full list in Table S1) for T_m and 272 descriptors (full list

in Table S2) for ΔH_m. Gradient Boosting Decision Trees (GBDT) algorithm from XGBoost library[S10] was used to build both (T_m and ΔH_m) ML-models. It is known that GBDT family of methods are one of the best ML method for tabular data[S11]. Each model hyperparameters were optimized with OPTUNA library[S12] in a nested 5-fold-cross-validation-loop. The performance of each model was measured in an outer loop of nested 5-fold-cross-validation leading to: mean absolute error (MAE) 32 $\pm$ 1 K and MAE 6.7$\pm$ 0.2 kJ/mol for T_m and ΔH_m, respectively (for details see Figures S4 and S5). Both models with the best hyperparameter set were refitted using full T_m and ΔH_m preprocessed datasets for the virtual screening.

Table S1: List of AlvaDesc's molecular descriptors used for building T_m ML-model

No.	Descriptor Name
1	Me
2	nAA
3	SCBO
4	RBN
5	RBF
6	nDB
7	nTB
8	nN
9	nO
10	nS
11	nF
12	nCL
13	nHM
14	nHet

Continued on next column/page

Table S1: *Continued*

No.	Descriptor Name
15	nX
16	H%
17	C%
18	N%
19	O%
20	Fsp3
21	max_conj_path
22	nCIC
23	Rbrid
24	MCD
25	NRS
26	NNRS
27	nR04
28	nR05
29	nR10
30	nBnz
31	ARR

Continued on next column/page

Table S1: *Continued*

No.	Descriptor Name
32	ZM1V
33	DBI
34	HNar
35	PJI2
36	PW3
37	PW5
38	MAXDN
39	MAXDP
40	Psi_i_A
41	LOC
42	PCR
43	X2A
44	AAC
45	IDDE
46	IVDE
47	rGes
48	IC1

Continued on next column/page

Table S1: *Continued*

No.	Descriptor Name
49	IC2
50	SIC0
51	SIC1
52	SIC2
53	SIC3
54	SpMax_A
55	SpMax_L
56	SpMAD_L
57	AVS_X
58	ChiA_X
59	WiA_D/Dt
60	J_D/Dt
61	SM1_Dz(Z)
62	SM1_Dz(v)
63	HyWi_B(m)
64	SpMax_B(m)
65	AVS_B(v)

Continued on next column/page

Table S1: *Continued*

No.	Descriptor Name
66	SpMax_B(v)
67	SpMAD_B(v)
68	SpPosA_B(e)
69	SpMax_B(s)
70	SpDiam_B(s)
71	ATSC1e
72	ATSC2e
73	ATSC3e
74	ATSC5e
75	ATSC6e
76	ATSC7e
77	ATSC8e
78	ATSC2s
79	MATS2m
80	MATS2v
81	MATS3v
82	MATS2e

Continued on next column/page

Table S1: *Continued*

No.	Descriptor Name
83	MATS2p
84	MATS4p
85	MATS2i
86	MATS2s
87	GATS1m
88	GATS2m
89	GATS1v
90	GATS1e
91	GATS2e
92	GATS5e
93	GATS1p
94	GATS1i
95	GATS7i
96	GATS8i
97	GATS1s
98	GATS2s
99	GATS3s

Continued on next column/page

Table S1: *Continued*

No.	Descriptor Name
100	JGI3
101	JGI5
102	JGI6
103	JGI7
104	JGI8
105	JGI9
106	JGI10
107	SpMax2_Bh(v)
108	SpMin1_Bh(v)
109	SpMin2_Bh(v)
110	SpMin1_Bh(s)
111	SpMin2_Bh(s)
112	P_VSA_LogP_2
113	P_VSA_LogP_3
114	P_VSA_LogP_5
115	P_VSA_LogP_8
116	P_VSA_MR_2

Continued on next column/page

Table S1: *Continued*

No.	Descriptor Name
117	P_VSA_MR_6
118	P_VSA_MR_7
119	P_VSA_m_2
120	P_VSA_m_3
121	P_VSA_m_4
122	P_VSA_v_2
123	P_VSA_v_3
124	P_VSA_e_2
125	P_VSA_e_3
126	P_VSA_i_2
127	P_VSA_i_4
128	P_VSA_s_3
129	P_VSA_s_4
130	P_VSA_s_6
131	P_VSA_ppp_P
132	P_VSA_ppp_A
133	P_VSA_ppp_con

Continued on next column/page

Table S1: *Continued*

No.	Descriptor Name
134	P_VSA_ppp_ter
135	P_VSA_charge_2
136	P_VSA_charge_3
137	P_VSA_charge_4
138	P_VSA_charge_5
139	P_VSA_charge_6
140	P_VSA_charge_9
141	P_VSA_charge_13
142	Eta_L_A
143	Eta_F_A
144	Eta_sh_y
145	Eta_D_AlphaB
146	Eta_epsi_5
147	Eta_D_epsiD
148	SpMAD_EA(bo)
149	SpMax_EA(dm)
150	SpAD_EA(dm)

Continued on next column/page

Table S1: *Continued*

No.	Descriptor Name
151	SpMax_EA(ri)
152	SpMax_AEA(bo)
153	SpMax_AEA(dm)
154	SM03_EA(dm)
155	SM09_AEA(dm)
156	SM11_AEA(dm)
157	SM12_AEA(ri)
158	Eig03_EA(dm)
159	Eig04_EA(dm)
160	Eig04_AEA(dm)
161	nCs
162	nCt
163	nCrs
164	nCrt
165	nCconj
166	nR=Cs
167	nR=Ct

Continued on next column/page

Table S1: *Continued*

No.	Descriptor Name
168	nArCOOH
169	nRCONHR
170	nArCONHR
171	nROCON
172	nArCO
173	nC(=N)N2
174	nArNH2
175	nArNHR
176	nArNR2
177	nRNHO
178	nN(CO)2
179	nOHp
180	nArOR
181	nRSR
182	nSO2N
183	nCRX3
184	nArX

Continued on next column/page

Table S1: *Continued*

No.	Descriptor Name
185	nPyrroles
186	nPyrazoles
187	nThiophenes
188	nOxazoles
189	nIsoxazoles
190	nPyridines
191	C-003
192	C-005
193	C-006
194	C-016
195	C-018
196	C-020
197	C-026
198	C-027
199	C-028
200	C-029
201	C-033

Continued on next column/page

Table S1: *Continued*

No.	Descriptor Name
202	C-034
203	C-035
204	C-039
205	C-040
206	C-041
207	C-043
208	C-044
209	H-047
210	H-049
211	H-051
212	H-052
213	H-053
214	O-058
215	O-060
216	N-067
217	N-069
218	N-070

Continued on next column/page

Table S1: *Continued*

No.	Descriptor Name
219	N-072
220	N-073
221	N-074
222	N-075
223	Cl-089
224	S-107
225	S-110
226	SssNH
227	SsssN
228	SdsN
229	SaasN
230	SaaNH
231	SdO
232	SssO
233	SaaO
234	SaaS
235	NssCH2

Continued on next column/page

Table S1: *Continued*

No.	Descriptor Name
236	NsssCH
237	NdssC
238	NaaaC
239	minsCH3
240	mindCH2
241	minssCH2
242	mindsCH
243	minaaCH
244	mindssC
245	minaasC
246	minssssC
247	mintN
248	minsOH
249	mindO
250	mindS
251	minsBr
252	MaxssCH2

Continued on next column/page

Table S1: *Continued*

No.	Descriptor Name
253	MaxaaCH
254	MaxdssC
255	MaxaasC
256	MaxddsN
257	CATS2D_01_DA
258	CATS2D_02_DA
259	CATS2D_04_DA
260	CATS2D_05_DA
261	CATS2D_06_DA
262	CATS2D_07_DP
263	CATS2D_02_DN
264	CATS2D_02_DL
265	CATS2D_03_DL
266	CATS2D_05_DL
267	CATS2D_06_DL
268	CATS2D_07_DL
269	CATS2D_09_DL

Continued on next column/page

Table S1: *Continued*

No.	Descriptor Name
270	CATS2D_00_AA
271	CATS2D_02_AA
272	CATS2D_04_AA
273	CATS2D_05_AA
274	CATS2D_06_AA
275	CATS2D_07_AA
276	CATS2D_08_AA
277	CATS2D_02_AP
278	CATS2D_02_AL
279	CATS2D_03_AL
280	CATS2D_04_AL
281	CATS2D_05_AL
282	CATS2D_06_AL
283	CATS2D_07_AL
284	CATS2D_02_PL
285	CATS2D_03_PL
286	CATS2D_07_PL

Continued on next column/page

Table S1: *Continued*

No.	Descriptor Name
287	CATS2D_08_PL
288	CATS2D_01_NL
289	CATS2D_02_NL
290	SHED_DA
291	SHED_DL
292	SHED_AN
293	SHED_LL
294	B01[C-N]
295	B01[C-O]
296	B01[C-I]
297	B01[N-N]
298	B01[N-O]
299	B02[C-O]
300	B02[N-N]
301	B02[N-O]
302	B02[N-S]
303	B02[N-Cl]

Continued on next column/page

Table S1: *Continued*

No.	Descriptor Name
304	B02[O-O]
305	B02[O-S]
306	B02[S-S]
307	B03[C-O]
308	B03[N-N]
309	B03[N-O]
310	B03[N-S]
311	B03[N-F]
312	B03[N-Cl]
313	B03[N-Br]
314	B03[O-O]
315	B03[O-S]
316	B03[O-F]
317	B03[O-Cl]
318	B03[O-Br]
319	B03[F-F]
320	B03[Cl-Cl]

Continued on next column/page

Table S1: *Continued*

No.	Descriptor Name
321	B04[C-C]
322	B04[C-O]
323	B04[N-N]
324	B04[N-O]
325	B04[N-S]
326	B04[N-F]
327	B04[N-Cl]
328	B04[O-O]
329	B04[O-S]
330	B04[O-F]
331	B04[O-Cl]
332	B04[S-Cl]
333	B04[F-F]
334	B04[Cl-Cl]
335	B05[C-C]
336	B05[C-N]
337	B05[C-O]

Continued on next column/page

Table S1: *Continued*

No.	Descriptor Name
338	B05[C-S]
339	B05[C-Br]
340	B05[N-N]
341	B05[N-O]
342	B05[N-S]
343	B05[N-F]
344	B05[N-Cl]
345	B05[N-Br]
346	B05[O-O]
347	B05[O-S]
348	B05[O-F]
349	B05[O-Cl]
350	B05[O-Br]
351	B05[S-Cl]
352	B06[C-C]
353	B06[C-N]
354	B06[C-O]

Continued on next column/page

Table S1: *Continued*

No.	Descriptor Name
355	B06[C-S]
356	B06[C-Br]
357	B06[N-N]
358	B06[N-O]
359	B06[N-S]
360	B06[N-F]
361	B06[N-Cl]
362	B06[N-Br]
363	B06[O-O]
364	B06[O-S]
365	B06[O-F]
366	B06[O-Cl]
367	B06[O-Br]
368	B06[S-Cl]
369	B07[C-C]
370	B07[C-N]
371	B07[C-O]

Continued on next column/page

Table S1: *Continued*

No.	Descriptor Name
372	B07[C-S]
373	B07[C-F]
374	B07[C-Cl]
375	B07[C-Br]
376	B07[N-N]
377	B07[N-O]
378	B07[N-S]
379	B07[N-F]
380	B07[N-Cl]
381	B07[N-Br]
382	B07[O-O]
383	B07[O-S]
384	B07[O-F]
385	B07[O-Cl]
386	B07[O-Br]
387	B08[C-C]
388	B08[C-N]

Continued on next column/page

Table S1: *Continued*

No.	Descriptor Name
389	B08[C-O]
390	B08[C-S]
391	B08[C-F]
392	B08[C-Cl]
393	B08[C-Br]
394	B08[N-N]
395	B08[N-O]
396	B08[N-S]
397	B08[N-F]
398	B08[N-Cl]
399	B08[O-O]
400	B08[O-S]
401	B08[O-F]
402	B08[O-Cl]
403	B08[O-Br]
404	B09[C-C]
405	B09[C-N]

Continued on next column/page

Table S1: *Continued*

No.	Descriptor Name
406	B09[C-O]
407	B09[C-S]
408	B09[C-F]
409	B09[C-Cl]
410	B09[C-Br]
411	B09[N-N]
412	B09[N-O]
413	B09[N-S]
414	B09[N-F]
415	B09[N-Cl]
416	B09[O-O]
417	B09[O-S]
418	B09[O-F]
419	B09[O-Cl]
420	B10[C-C]
421	B10[C-N]
422	B10[C-O]

Continued on next column/page

Table S1: *Continued*

No.	Descriptor Name
423	B10[C-S]
424	B10[C-F]
425	B10[C-Cl]
426	B10[C-Br]
427	B10[N-N]
428	B10[N-O]
429	B10[N-S]
430	B10[N-F]
431	B10[N-Cl]
432	B10[O-O]
433	B10[O-S]
434	B10[O-F]
435	B10[O-Cl]
436	F01[C-O]
437	F02[C-N]
438	F02[C-O]
439	F03[C-N]

Continued on next column/page

Table S1: *Continued*

No.	Descriptor Name
440	F03[C-O]
441	F04[C-N]
442	F04[C-O]
443	F05[C-N]
444	F05[C-O]
445	F06[C-N]
446	F06[C-O]
447	F07[C-N]
448	F07[C-O]
449	F08[C-N]
450	TPSA(Tot)
451	SAscore
452	Ro5
453	DLS_01
454	DLS_02
455	DLS_03
456	DLS_04

Continued on next column/page

Table S1: *Continued*

No.	Descriptor Name
457	DLS_05
458	DLS_06
459	DLS_07
460	DLS_cons
461	LLS_01
462	LLS_02
463	QEDu
464	MDEN-23
465	ringChiralCenter
466	meanDistFromCC
467	phLevel1
468	phLevel2
469	phLevel3
470	arLevel1
471	arLevel2
472	s2_phSize
473	s3_phSize

Continued on next column/page

Table S1: *Continued*

No.	Descriptor Name
474	s1_phRelSize_2
475	s2_numAroBonds
476	s3_numAroBonds

Table S2: List of AlvaDesc's molecular descriptors used for building ΔH_m ML-model

No.	Descriptor Name
1	Se
2	Me
3	nAA
4	RBN
5	nTB
6	nN
7	nHM
8	H%
9	C%
10	O%
11	X%
12	nCsp3
13	Fsp3
14	max_conj_path

Continued on next column/page

Table S2: *Continued*

No.	Descriptor Name
15	nCIC
16	MCD
17	RCI
18	NRS
19	NNRS
20	nR05
21	ARR
22	D/Dtr06
23	S3K
24	PW3
25	PW4
26	MAXDN
27	MAXDP
28	LOC
29	AAC
30	IVDE
31	Yindex

Continued on next column/page

Table S2: *Continued*

No.	Descriptor Name
32	IC1
33	IC2
34	SIC0
35	SIC1
36	SIC2
37	SIC3
38	CIC5
39	MATS1i
40	GATS1m
41	GATS2m
42	GATS5m
43	GATS1e
44	GATS2e
45	GATS5e
46	GATS8e
47	GATS1p
48	GATS2p

Continued on next column/page

Table S2: *Continued*

No.	Descriptor Name
49	GATS5p
50	GATS1i
51	GATS2i
52	GATS3i
53	GATS4i
54	GATS5i
55	GATS6i
56	GATS7i
57	GATS8i
58	JGI1
59	JGI2
60	JGI3
61	JGI4
62	JGI5
63	JGI6
64	JGI7
65	JGI8

Continued on next column/page

Table S2: *Continued*

No.	Descriptor Name
66	JGI9
67	JGI10
68	SpMax1_Bh(m)
69	SpMax1_Bh(e)
70	SpMin1_Bh(e)
71	P_VSA_LogP_2
72	P_VSA_MR_5
73	P_VSA_m_2
74	P_VSA_m_4
75	P_VSA_v_3
76	P_VSA_e_2
77	P_VSA_charge_3
78	P_VSA_charge_6
79	P_VSA_charge_8
80	P_VSA_charge_10
81	nCs
82	nCt

Continued on next column/page

Table S2: *Continued*

No.	Descriptor Name
83	nCrt
84	nR=Cp
85	nRCOOH
86	nArCOOR
87	nRCO
88	nArCO
89	nCONN
90	nArC=N
91	nArCN
92	nN(CO)2
93	nArOR
94	C-005
95	C-025
96	C-028
97	C-037
98	C-042
99	H-046

Continued on next column/page

Table S2: *Continued*

No.	Descriptor Name
100	H-049
101	H-052
102	N-073
103	N-074
104	N-075
105	SssCH2
106	NaasC
107	NdsN
108	minsCH3
109	minssCH2
110	mindsCH
111	minaaCH
112	minaasC
113	minsNH2
114	minssNH
115	mintN
116	minaasN

Continued on next column/page

Table S2: *Continued*

No.	Descriptor Name
117	minsOH
118	mindO
119	minssO
120	minaaO
121	mindS
122	minssS
123	minaaS
124	minsF
125	minsCl
126	minsBr
127	MaxaasC
128	MaxaaaC
129	CATS2D_02_DD
130	CATS2D_02_DA
131	CATS2D_05_DL
132	CATS2D_02_AL
133	CATS2D_03_AL

Continued on next column/page

Table S2: *Continued*

No.	Descriptor Name
134	CATS2D_04_AL
135	CATS2D_01_NL
136	SHED_DA
137	SHED_DL
138	SHED_AA
139	SHED_AL
140	SHED_NL
141	B01[C-C]
142	B01[C-O]
143	B01[C-S]
144	B01[N-N]
145	B01[N-O]
146	B01[N-S]
147	B01[O-S]
148	B02[C-C]
149	B02[C-O]
150	B02[C-S]

Continued on next column/page

Table S2: *Continued*

No.	Descriptor Name
151	B02[N-N]
152	B02[N-O]
153	B02[N-S]
154	B02[O-O]
155	B02[F-F]
156	B02[Cl-Cl]
157	B03[C-C]
158	B03[N-N]
159	B03[N-O]
160	B03[N-S]
161	B03[N-Cl]
162	B03[O-O]
163	B03[O-S]
164	B03[O-F]
165	B03[O-Cl]
166	B03[F-F]
167	B03[Cl-Cl]

Continued on next column/page

Table S2: *Continued*

No.	Descriptor Name
168	B04[C-C]
169	B04[N-N]
170	B04[N-O]
171	B04[N-S]
172	B04[N-Cl]
173	B04[O-O]
174	B04[O-S]
175	B04[O-F]
176	B04[O-Cl]
177	B04[F-F]
178	B04[Cl-Cl]
179	B05[C-C]
180	B05[C-N]
181	B05[C-O]
182	B05[C-S]
183	B05[C-F]
184	B05[C-Cl]

Continued on next column/page

Table S2: *Continued*

No.	Descriptor Name
185	B05[C-Br]
186	B05[N-N]
187	B05[N-O]
188	B05[N-S]
189	B05[N-Cl]
190	B05[O-O]
191	B05[O-S]
192	B05[O-F]
193	B05[O-Cl]
194	B05[F-F]
195	B05[Cl-Cl]
196	B06[C-C]
197	B06[C-N]
198	B06[C-O]
199	B06[C-S]
200	B06[C-F]
201	B06[C-Cl]

Continued on next column/page

Table S2: *Continued*

No.	Descriptor Name
202	B06[C-Br]
203	B06[N-N]
204	B06[N-O]
205	B06[N-Cl]
206	B06[O-O]
207	B06[O-F]
208	B06[O-Cl]
209	B07[C-C]
210	B07[C-N]
211	B07[C-O]
212	B07[C-S]
213	B07[C-F]
214	B07[C-Cl]
215	B07[C-Br]
216	B07[N-N]
217	B07[N-O]
218	B07[N-Cl]

Continued on next column/page

Table S2: *Continued*

No.	Descriptor Name
219	B07[O-O]
220	B07[O-F]
221	B07[O-Cl]
222	B07[Cl-Cl]
223	B08[C-C]
224	B08[C-N]
225	B08[C-O]
226	B08[C-S]
227	B08[C-Cl]
228	B08[C-Br]
229	B08[N-N]
230	B08[N-O]
231	B08[O-O]
232	B08[O-F]
233	B09[C-C]
234	B09[C-N]
235	B09[C-O]

Continued on next column/page

Table S2: *Continued*

No.	Descriptor Name
236	B09[C-S]
237	B09[C-Cl]
238	B09[C-Br]
239	B09[N-N]
240	B09[N-O]
241	B09[O-O]
242	B09[O-F]
243	B10[C-C]
244	B10[C-N]
245	B10[C-O]
246	B10[C-S]
247	B10[C-F]
248	B10[C-Cl]
249	B10[N-N]
250	B10[N-O]
251	B10[O-O]
252	MLOGP

Continued on next column/page

Table S2: *Continued*

No.	Descriptor Name
253	Ro5
254	DLS_01
255	DLS_02
256	DLS_03
257	DLS_04
258	DLS_05
259	DLS_06
260	DLS_07
261	DLS_cons
262	LLS_01
263	LLS_02
264	QEDu
265	meanDistFromCC
266	phLevel1
267	phLevel2
268	arLevel3
269	s2_phRelSize

Continued on next column/page

Table S2: *Continued*

No.	Descriptor Name
270	s3_phRelSize
271	s2_pathLength
272	s4_numSharedNeighbors

SI-1.3 Virtual screening

A virtual chemical library (462,000 unique molecules) was formed by a combination of the United States Patent and Trademark Office (USPTO) patent records and catalogs of chemical vendors. Molecules from patents are potentially useful for their applicability and potential synthesizability. Vendors include TCI America (51k mols, accessed October 2021) and Sigma-Aldrich (233k mols, accessed October 2021). The initial virtual library was pre-screened for the following chemical properties: the number of conjugated rings (RingCount $\geq$ 3), the molecular weight (M.W. $\geq$ 300), the aromatic proportion (A.P. $\geq$ 0.8), and the number of rotatable bonds (NumRotableBonds $\geq$ 3). The resulting focused library of 7742 molecules reflects the relevant chemical space of primarily aromatic molecules. All properties were computed via RDKit library[S13]; Aromatic Proportion is defined as the ratio of Heavy Atom Count in an Aromatic State to total Heavy Atom Count. For each molecule in the focused library, ΔH_m^{pred} and T_m^{pred} were predicted, and ΔG_c^{pred} was computed according to Equation 1. Molecules with $T_m^{pred} \geq 550$ K and $\Delta G_c^{pred} <$ -7.5 kJ/mol were selected according to the heuristic rule of the crystallization driving force at T_c as a function of T_m for the materials developed previously[S14]. That led to 44 candidate molecules (see Table S3) suitable for expert assessment.

SI-1.4 t-SNE analysis

Focused library, ML library (see Figure 1) and previously[S14] characterized materials were featurized by computing descriptors used for ML-modelling of T_m and ΔH_m (full list of descriptors provided in Table S1 and Table S2)) available through AlvaDesc 2.0[S8]. Then each descriptor was scaled individually via MinMax scaler and t-SNE 2D projection was done using the Scikit-learn library[S9].

SMILES	INCHIKEY	Name	Characterization	Ref
C1=CC=C(C=C1)C1=C2C=CC=CC2=C(C2=CC=CC=C2)C2=C(C3=CC=CC=C3)C3=C(C=CC=C3)C(=C12)C1=CC=CC=C1	YYMBJDOZVAITBP-UHFFFAOYSA-N	rubrene	Platelet Forming	dull2020
C1=CC=C(C=C1)C1=CC=C(C=C1)N1C2=C(C=CC=C2)C2=C3N(C4=CC=C(C=C4)C4=CC=CC=C4)C4=CC=CC=C4C3=CC=C12	SPDDAJDOPMIIAO-UHFFFAOYSA-N			
O1C2=C(OC3=CC=C(C=C13)N(C1=CC=CC=C1)C1=C3C=CC=CC3=CC=C1)C=C(C=C2)N(C1=CC=CC=C1)C1=CC=CC2=CC=CC=C12	HRPBWJYAXSSXJK-UHFFFAOYSA-N			
O1C2=C(C=C1C1=CC=C(C=CC3=CC=C(C=C3)C3=CC=CC=C3)C=C1)C=C(C=C2)C1=CC=CC=C1	FAZCYGQGRLYIQP-UHFFFAOYSA-N			
N(C1=CC=C(C=C1)C1=CC=CC=C1)C1=NC=CC(=N1)C1=CC=C(C=C1)C1=CC=C(C=C1)N(C1=CC=CC=C1)C1=CC=CC=C1	UVQNVCCVBLJSEM-UHFFFAOYSA-N			
N(C1=CC2=CC=CC=C2C=C1)C1=NC(=CC=N1)C1=CC=C(C=C1)C1=CC=C(C=C1)N(C1=CC=CC=C1)C1=CC=CC=C1	WCGMZXRESBNDJG-UHFFFAOYSA-N			
N(C1=CC=CC=C1)C1=NC=CC(=N1)C1=CC=C(C=C1)C1=CC=C(C=C1)N(C1=CC=CC=C1)C1=CC=CC=C1	WMIXUWVRSSUJEJ-UHFFFAOYSA-N			
OC1=CC=C(C(=C1)O)C1=NN=NC(=C1C1=CC=C(O)C=C1O)C1=CC=CC2=CC=CC=C12	ASWVRBKLTUIGGT-UHFFFAOYSA-N			
CC1=CC=C(C=C1)N1C(=CC=C1C1=CC=C(C=CC2=CC=CC=C2)C=C1)C1=CC=C(C=CC2=CC=CC=C2)C=C1	GEWIZGAXHZJANN-UHFFFAOYSA-N			
C1C=C(C=CC1(N1N=C(C=C1C1=CC=CC=C1)C1=CC2=CC=CC=C2C=C1)N1N=C(C=C1C1=CC=CC=C1)C1=CC2=C(C=CC=C2)C=C1)C1=CC=CC=C1	JPFGYTWFUJOSLA-UHFFFAOYSA-N			
OC(=O)C1=CC=C(COC2=CC=C3C=C(C=CC3=C2)C2=CC=C(N2C2=CC=CC=C2)C2=CC=CC=C2)C=C1	KQALUCCAFFZUBB-UHFFFAOYSA-N			
C1=CC=C(C=C1)P(C1=CC=CC=C1)C1=CC=C2C=CC=CC2=C1C1=C(C=CC2=C1C=CC=C2)P(C1=CC=CC=C1)C1=CC=CC=C1	MUALRAIOVNYAIW-UHFFFAOYSA-N	rac-BINAP	Platelet Forming	Johnson2024
C1=CC=C(C=C1)C1=CC=C(C=C1)C1=NC(=NC(=N1)C1=CC=C(C=C1)C1=CC=CC=C1)C1=CC=C(C=C1)C1=CC=CC=C1	CENPSTJGQOQKKW-UHFFFAOYSA-N	TBT	Platelet Forming	Johnson2024
O1C(=NN=C1C1=CC=CC=C1C1=CC=CC=C1)C1=CC=C(C=CC2=CC=C(C=C2)N2N=CC(=N2)C2=CC=CC=C2)C=C1	LSZSSMIRECJENG-UHFFFAOYSA-N			
c1csc(-c2ccc(-c3ccc(-c4ccc(-c5ccc(-c6ccc(-c7ccc(-c8cccs8)s7)s6)s5)s4)s3)s2)c1	GCMCTPRNKVKGCY-UHFFFAOYSA-N			
c1csc(-c2ccc(-c3ccc(-c4ccc(-c5ccc(-c6ccc(-c7cccs7)s6)s5)s4)s3)s2)c1	DCIYLINCGGGNLC-UHFFFAOYSA-N			
c1csc(-c2ccc(-c3ccc(-c4ccc(-c5ccc(-c6cccs6)s5)s4)s3)s2)c1	KUJYDIFFRDAYDH-UHFFFAOYSA-N	alpha-Sexithiophene		
Brc1ccc(-c2ccc(-c3ccc(-c4ccc(-c5ccc(Br)s5)s4)s3)s2)s1	IEDJCFHSALKQRZ-UHFFFAOYSA-N			
c1ccc(-c2c(-c3ccccc3)c(-c3ccccc3)c3c4c(-c5ccccc5)c5c(-c6ccccc6)c(-c6ccccc6)c(-c6ccccc6)c-5c=4c(-c4ccccc4)c2-3)cc1	ZEVFDNVCEKXSEV-UHFFFAOYSA-N			
c1ccc(-c2ccc(-c3ccc(-c4ccc(-c5ccc(-c6ccccc6)cc5)s4)s3)cc2)cc1	SJNXLFCTPWBYSE-UHFFFAOYSA-N			
c1ccc(-c2oc3cc4c(-c5ccc(-n6c7ccccc7c7ccccc76)cc5)c(-c5ccccc5)oc4cc3c2-c2ccc(-n3c4ccccc4c4ccccc43)cc2)cc1	FHDKOSLORKUETK-UHFFFAOYSA-N	CZBDF	Resists Crystallization	Johnson2024
c1ccc(-c2ccc(-c3ccc([Si](c4ccccc4)(c4ccc(-c5ccc(-c6ccccc6)cc5)cc4)c4ccc(-c5ccc(-c6ccccc6)cc5)cc4)cc3)cc2)cc1	KFWDFMXZMGBRLO-UHFFFAOYSA-N			
Brc1ccc(-c2nc(-c3ccccc3)c(-c3ccc(-c4[nH]c(-c5ccc(Br)cc5)nc4-c4ccccc4)cc3)[nH]2)cc1	BFOWTSPHCWRSIH-UHFFFAOYSA-N			
O=c1oc2ccc3ccccc3c2cc1-c1cnc(Nc2ccc(Cl)c(Cl)c2)s1	HMUCXBLRFMQQBA-UHFFFAOYSA-N			
C1=Cc2nc1c(-c1ccncc1)c1ccc([nH]1)c(-c1ccncc1)c1nc(c(-c3ccncc3)c3ccc([nH]3)c2-c2ccncc2)C=C1	DNZSHSJERXNJGX-UHFFFAOYSA-N			
c1csc(-c2ccc(-c3ccc(-c4ccc(-c5cccs5)s4)s3)s2)c1	YFBLUJZFRBFQMR-UHFFFAOYSA-N			
Oc1ccc(-c2c3nc(c(-c4ccc(O)cc4)c4ccc([nH]4)c(-c4ccc(O)cc4)c4nc(c(-c5ccc(O)cc5)c5ccc2[nH]5)C=C4)C=C3)cc1	VFHDWGAEEDVVPD-UHFFFAOYSA-N			
Nc1c(S(=O)(=O)c2ccccc2)c2nc3ccccc3nc2n1-c1ccc(F)cc1	CSFVFDHRYKBBPD-UHFFFAOYSA-N			
O=c1[nH]c2ccccc2n1C1CCN(Cc2ccc(-c3nc4cc5[nH]cnc5cc4nc3-c3ccccc3)cc2)CC1	BIWGYFZAEWGBAL-UHFFFAOYSA-N			
O=C(O)c1ccc(-c2c3nc(c(-c4ccccc4)c4ccc([nH]4)c(-c4ccccc4)c4nc(c(-c5ccccc5)c5ccc2[nH]5)C=C4)C=C3)cc1	GZTMFXHPFNRUNU-UHFFFAOYSA-N			
c1ccc2c(c1)c1ccccc1n2-c1cc(-n2c3ccccc3c3ccccc32)cc(-n2c3ccccc3c3ccccc32)c1	DVNOWTJCOPZGQA-UHFFFAOYSA-N	TCP	Spherulite Forming	dull2020
c1ccc2c(c1)c1ccccc1n2-c1ccc(-c2cc(-c3ccc(-n4c5ccccc5c5ccccc54)cc3)cc(-c3ccc(-n4c5ccccc5c5ccccc54)cc3)c2)cc1	XCICDYGIJBPNPC-UHFFFAOYSA-N	TPB-Cz	Spherulite Forming	Johnson2024
c1ccc(-n2c3ccccc3c3cc(-c4ccc5c(c4)[nH]c4cc(-c6ccc7c(c6)c6ccccc6n7-c6ccccc6)ccc45)ccc32)cc1	OXYUAOUFXFTZFU-UHFFFAOYSA-N	9DT	Resists Crystallization	Johnson2024
c1ccc(-n2c(-c3ccc(-c4ccc5c(c4)c4cc(-c6ccc(-c7nc8ccccc8n7-c7ccccc7)cc6)ccc4n5-c4ccccc4)cc3)nc3ccccc32)cc1	CWUOZRFIMAVZLO-UHFFFAOYSA-N			
c1ccc(N(c2ccccc2)c2ccc3c(c2)C2(c4cc(N(c5ccccc5)c5ccccc5)ccc4-3)c3cc(N(c4ccccc4)c4ccccc4)ccc3-c3ccc(N(c4ccccc4)c4ccccc4)cc32)cc1	MQRCTQVBZYBPQE-UHFFFAOYSA-N	Spiro-TAD	Platelet Forming	Johnson2024
O=C(O)c1ccc(-c2nc(-c3ccccc3)c(-c3ccccc3)[nH]2)cc1	BCXPNUSETAZHEQ-UHFFFAOYSA-N			
Oc1ccc(C(c2ccccc2)(c2ccccc2)c2ccc(O)cc2)cc1	BATCUENAARTUKW-UHFFFAOYSA-N			
c1ccc2c(c1)c1ccccc1n2-c1ccc(N(c2ccc(-n3c4ccccc4c4ccccc43)cc2)c2ccc(-n3c4ccccc4c4ccccc43)cc2)cc1	AWXGSYPUMWKTBR-UHFFFAOYSA-N	TCTA	Platelet Forming	dull2020
O=[N+]([O-])c1ccc(-c2nc(-c3ccncc3)c(-c3ccc(F)cc3)[nH]2)cc1	BGIYKDUASORTBB-UHFFFAOYSA-N			
c1csc(-c2ccc(-c3ccc(N(c4ccc(-c5ccc(-c6cccs6)cc5)cc4)c4ccc(-c5ccc(-c6cccs6)cc5)cc4)cc3)cc2)c1	STWJEWBEHUGXTL-UHFFFAOYSA-N			
c1ccc(-c2ccc(-c3ccc(-c4ccc(-c5ccc(-c6ccccc6)cc5)cc4)cc3)cc2)cc1	ZEMDSNVUUOCIED-UHFFFAOYSA-N	p-Sexiphenyl		
Cc1[nH]n(-c2ccccc2)c(=O)c1-c1c(C)[nH]n(-c2ccccc2)c1=O	CIJVXSIRUQCTBV-UHFFFAOYSA-N			
Nc1ccc(-c2c3nc(c(-c4ccc(N)cc4)c4ccc([nH]4)c(-c4ccc(N)cc4)c4nc(c(-c5ccc(N)cc5)c5ccc2[nH]5)C=C4)C=C3)cc1	REPFNYFEIOZRLM-UHFFFAOYSA-N			
Brc1ccc2c(c1)c1cc(-c3ccc4c(c3)c3cc(Br)ccc3n4-c3ccccc3)ccc1n2-c1ccccc1	VTIPHPNQFUCDAW-UHFFFAOYSA-N			

Table S3: List of 44 molecules in Focused ML Library with their functional annotation.

SI-1.5 Materials

The materials used in this work were purchased from commercial vendors and used as received. From Alfa Aesar: 2,2'-bis(diphenylphosphino)-1,1'-binaphthyl (rac-BINAP). From Lumtec: 4,4',4-tris(carbazol-9-yl)triphenylamine (TcTa). From Sigma-Aldrich: N,N,N',N'-tetrakis(4-methoxyphenyl)benzidine (MeO-TPD). From TCI: 2,4,6-tri([1,1'-biphenyl]-4-yl)-1,3,5-triazine (TBT), 2,2',7,7'-tetrakis(N,N-diphenylamino)-9,9-spirobifluorene (spiro-TAD), 1,3,5-tris[4-(9-carbazolyl)phenyl]benzene (TPB-Cz), 9,9"-diphenyl-9H,9'H,9"H-3,2':7',3"-tercarbazole (9DT), and 9-[4-[3-(4-carbazol-9-ylphenyl)-2,6-diphenylfuro[2,3-f][1]benzofuran-7-yl]phenyl]carbazole (CZBDF). The materials TcTa and MeO-TPD were utilized as organic underlayers that aided the crystallization of spiro-TAD and TBT, respectively.

SI-1.6 Fabrication

Materials were deposited onto glass/indium tin oxide (ITO) substrates that were cleaned via successive deionized water, acetone, and isopropyl alcohol sonication followed by an oxygen plasma etch. The materials were deposited via vacuum thermal evaporation at approximately 1x10^{-6} Torr. Several variables - film thickness, annealing temperature, annealing time, and the inclusion of an organic underlayer - were tuned for each molecule to optimize the morphology, size, and coverage of crystals in the organic film. These variables are reported in Table S4. Rac-BINAP, TPB-Cz, 9DT, and CZBDF were deposited directly onto the glass/ITO substrates. In the case of TBT and spiro-TAD, a thin organic underlayer of 5 nm was deposited between the substrate and the material being investigated to aid the crystallization process;[S15] MeO-TPD was used for TBT, and TcTa was used for spiro-TAD. These materials were chosen based on their glass transition temperatures,[S14] which complemented the thermal properties of the materials being investigated in this work as determined by DSC. The thin films were crystallized via post-deposition annealing, using a pre-heated hotplate in a nitrogen glovebox. Ellipsometry was performed at atmosphere on amorphous, un-annealed films, while post-annealed substrates were exposed to atmosphere to perform

POM and XRD measurements. There was no exposure to atmosphere between deposition and annealing.

SI-1.7 Equipment and characterization

Images of the annealed crystalline films were taken with a polarized optical microscope (POM), model Olympus BX60F5. Ellipsometry was performed using a J.A. Woollam M2000 variable angle spectroscopic ellipsometer and the resulting data was analyzed using CompleteEASE software; this technique was employed for tooling the thermal evaporator and determining thin film thickness. The empirical thermal properties of the organics studied here were obtained using a TA Instruments differential scanning calorimeter (DSC) 2500 equipped with an RCS90 cooler. The furnace chamber of the DSC was continuously flushed with nitrogen. For each material, approximately 5 mg of the as-received materials were loaded into a hermetically-sealed, aluminum pan with a small hole on the sealed lid; the reference pan also had a small hole to match the conditions of the sample pan. The DSC was calibrated for temperature using a melting temperature measurement for indium and for heat capacity using sapphire standards. For the secondary DSC scan of CZBDF seen in Figure S3, a PerkinElmer DSC-8500 Differential Scanning Calorimeter was used with an empty aluminum pan as reference. The scan rate for all DSC scans was 10°C/min. The thermal properties obtained by DSC reported here were taken at the onset values of each thermal event. X-ray diffraction (XRD) was performed using a Bruker D8 Discover X-ray diffractometer with a copper source, wavelength 1.54 Å.

Table S4: A summary of the optimized fabrication conditions for each crystallized material in this work. All underlayer materials are 5 nm thick and deposited between the ITO and the organic film to be crystallized.

	Molecule	Annealing temperature (°C)	Annealing time (min)	Film thickness (nm)	Underlayer
a	rac-BINAP	140	5	60	–
b	TBT	160	0.33	30	MeO-TPD
c	spiro-TAD	200	5	60	TcTa
d	TPB-Cz	200	5	38	–

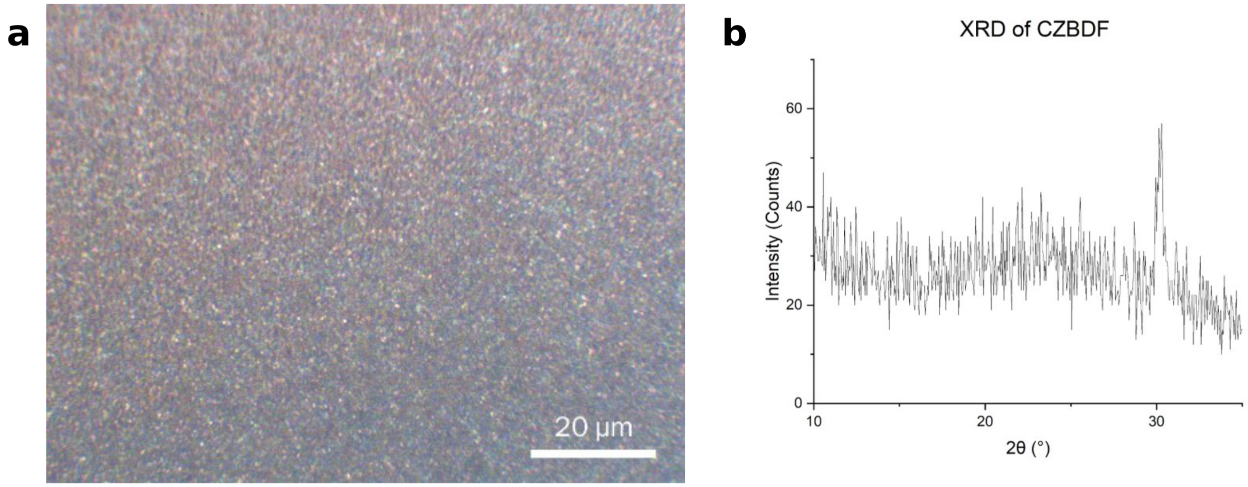

Figure S1: (a) POM image of CZBDF after post-deposition annealing and (b) XRD of CZBDF grown on ITO with post-deposition annealing. Scan is featureless with the exception of a peak at $2\theta = 30.2°$ from the ITO substrate.

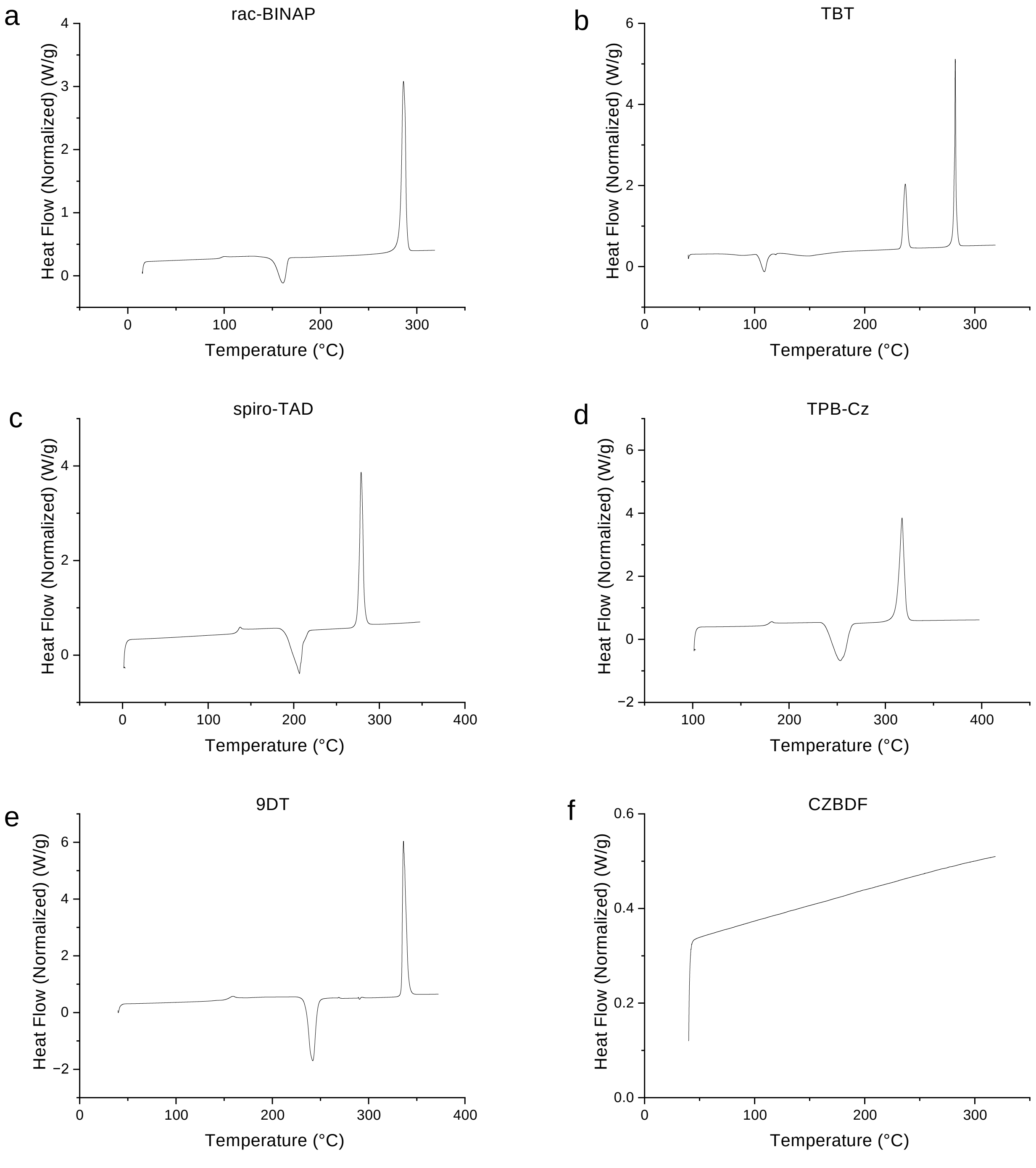

Figure S2: DSC scans of the six materials in this work. The thermal characteristics of each material were taken at the onset values of each thermal event.

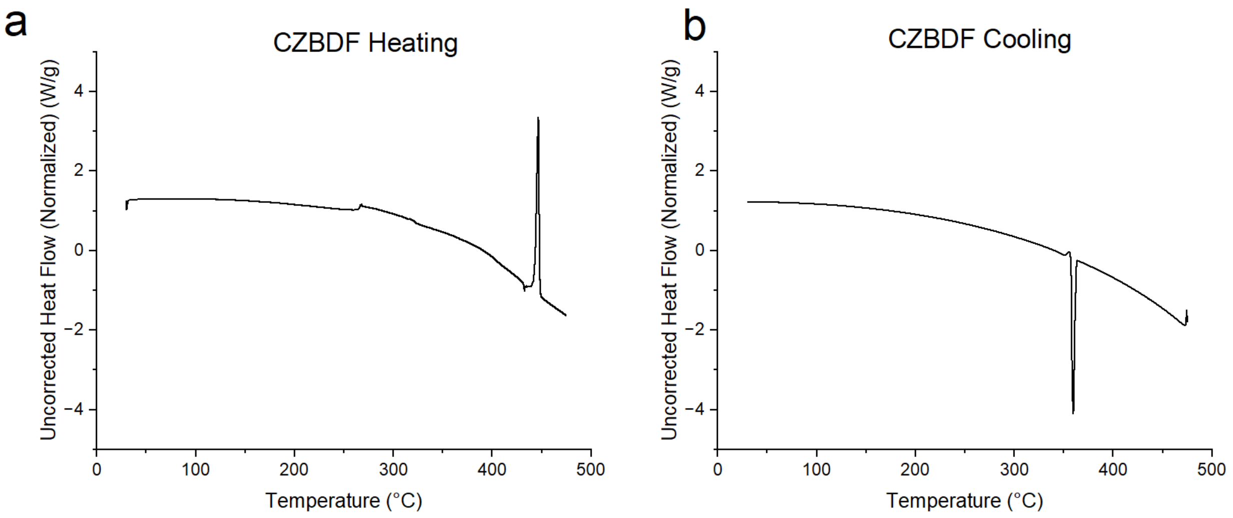

Figure S3: DSC scan of CZBDF showing initial heating (a) and the following cooling (b). A glass transition and melting peak are observed upon heating, and a crystallization peak is seen upon cooling.

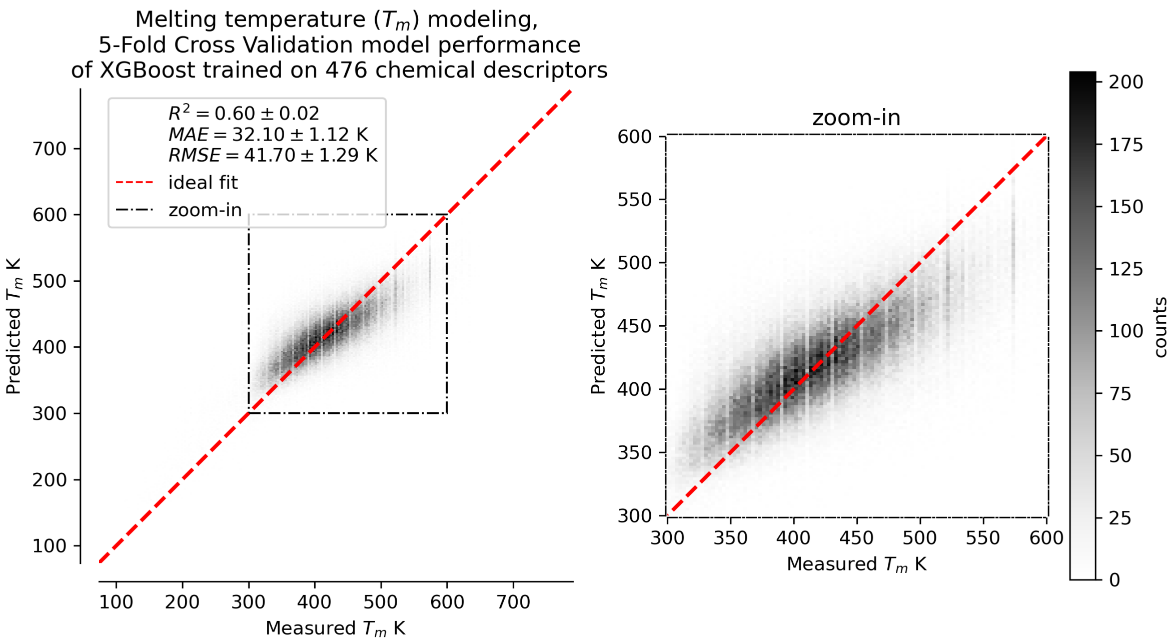

Figure S4: T_m ML model performance measured over 5-fold Cross-Validation.

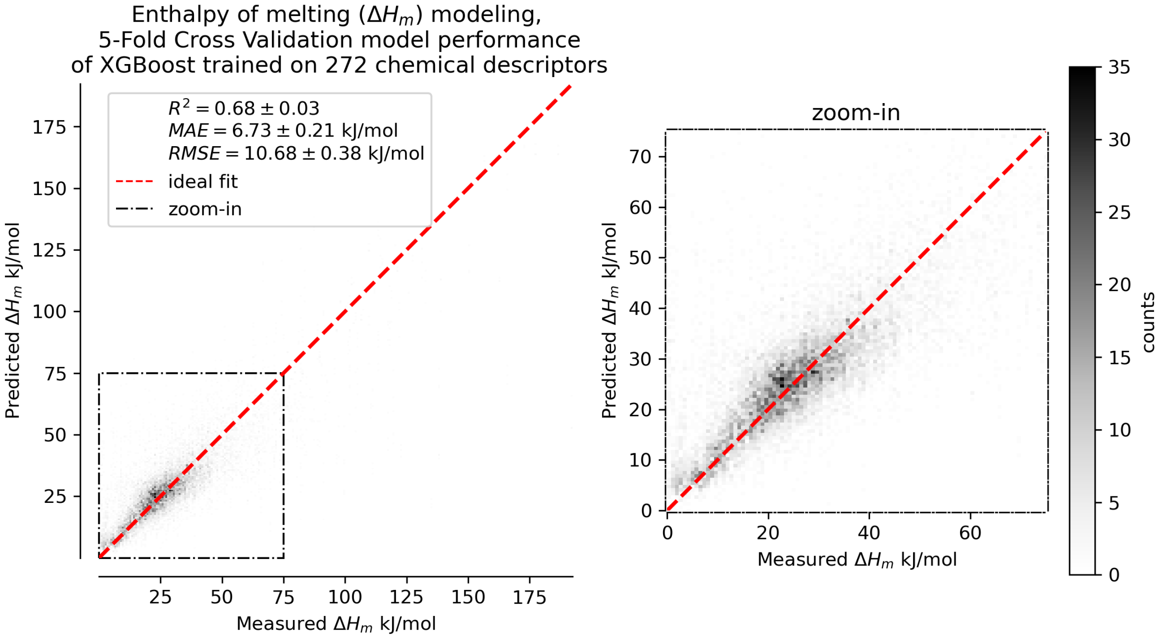

Figure S5: ΔH_m ML model performance measured over 5-fold Cross-validation.

References

(S1) Tetko, I. V.; M. Lowe, D.; Williams, A. J. The development of models to predict melting and pyrolysis point data associated with several hundred thousand compounds mined from PATENTS. *Journal of Cheminformatics* **2016**, *8*, 2, DOI: 10.1186/s13321-016-0113-y.

(S2) Tetko, I. V.; Sushko, Y.; Novotarskyi, S.; Patiny, L.; Kondratov, I.; Petrenko, A. E.; Charochkina, L.; Asiri, A. M. How Accurately Can We Predict the Melting Points of Drug-like Compounds? *Journal of Chemical Information and Modeling* **2014**, *54*, 3320–3329, DOI: 10.1021/ci5005288.

(S3) Acree, W.; Chickos, J. S. Phase Transition Enthalpy Measurements of Organic and Organometallic Compounds. Sublimation, Vaporization and Fusion Enthalpies From 1880 to 2015. Part 1. C_1 - C_{10}. *Journal of Physical and Chemical Reference Data* **2016**, *45*, 033101, DOI: 10.1063/1.4948363.

(S4) Acree, W.; Chickos, J. S. Phase Transition Enthalpy Measurements of Organic and Organometallic Compounds and Ionic Liquids. Sublimation, Vaporization, and Fusion Enthalpies from 1880 to 2015. Part 2. C11–C192. *Journal of Physical and Chemical Reference Data* **2017**, *46*, 013104, DOI: 10.1063/1.4970519.

(S5) Acree, W.; Chickos, J. S. Phase Transition Enthalpy Measurements of Organic Compounds. An Update of Sublimation, Vaporization, and Fusion Enthalpies from 2016 to 2021. *Journal of Physical and Chemical Reference Data* **2022**, *51*, 043101, DOI: 10.1063/5.0081916.

(S6) Fourches, D.; Muratov, E.; Tropsha, A. Trust, but Verify II: A Practical Guide to Chemogenomics Data Curation. *Journal of Chemical Information and Modeling* **2016**, *56*, 1243–1252, DOI: 10.1021/acs.jcim.6b00129.

(S7) MolVS. https://github.com/mcs07/MolVS.

(S8) Mauri, A. In *Ecotoxicological QSARs*; Roy, K., Ed.; Springer US: New York, NY, 2020; pp 801–820, DOI: 10.1007/978-1-0716-0150-1_32, Series Title: Methods in Pharmacology and Toxicology.

(S9) Pedregosa, F.; Varoquaux, G.; Gramfort, A.; Michel, V.; Thirion, B.; Grisel, O.; Blondel, M.; Prettenhofer, P.; Weiss, R.; Dubourg, V.; Vanderplas, J.; Passos, A.; Cournapeau, D.; Brucher, M.; Perrot, M.; Duchesnay, Scikit-learn: Machine Learning in Python. *Journal of Machine Learning Research* **2011**, *12*, 2825–2830.

(S10) Chen, T.; Guestrin, C. XGBoost: A Scalable Tree Boosting System. Proceedings of the 22nd ACM SIGKDD International Conference on Knowledge Discovery and Data Mining. San Francisco California USA, 2016; pp 785–794, DOI: 10.1145/2939672.2939785.

(S11) Shwartz-Ziv, R.; Armon, A. Tabular data: Deep learning is not all you need. *Information Fusion* **2022**, *81*, 84–90, DOI: 10.1016/j.inffus.2021.11.011.

(S12) Akiba, T.; Sano, S.; Yanase, T.; Ohta, T.; Koyama, M. Optuna: A Next-generation Hyperparameter Optimization Framework. Proceedings of the 25th ACM SIGKDD International Conference on Knowledge Discovery & Data Mining. Anchorage AK USA, 2019; pp 2623–2631, DOI: 10.1145/3292500.3330701.

(S13) RDKit. https://www.rdkit.org.

(S14) Dull, J. T.; Wang, Y.; Johnson, H.; Shayegan, K.; Shapiro, E.; Priestley, R. D.; Geerts, Y. H.; Rand, B. P. Thermal Properties, Molecular Structure, and Thin-Film Organic Semiconductor Crystallization. *The Journal of Physical Chemistry C* **2020**, *124*, 27213–27221, DOI: 10.1021/acs.jpcc.0c09408.

(S15) Fusella, M. A.; Yang, S.; Abbasi, K.; Choi, H. H.; Yao, Z.; Podzorov, V.; Avishai, A.; Rand, B. P. Use of an Underlayer for Large Area Crystalliza-

tion of Rubrene Thin Films. *Chemistry of Materials* **2017**, *29*, 6666–6673, DOI: 10.1021/acs.chemmater.7b01143.
